# Estimated daily intake of epichlorohydrin and certain heavy metals of bagged and loose black teas

**DOI:** 10.1007/s13197-022-05652-5

**Published:** 2022-12-06

**Authors:** Suheir M. F. Nour, Amany M. M. El-Desoky, Nagla A. Hassan, Khaled A. Osman

**Affiliations:** 1 Department of Economic Home, Faculty of Agriculture, Alexandria University, P.O Box 21545, Al-Shatby, Alexandria, Egypt; 2 Department of Pesticide Chemistry and Technology, Faculty of Agriculture, Alexandria University, Aflatoon St., P.O Box 21545, EL-Shatby, Alexandria, 21545 Egypt

**Keywords:** Tea bags, Epichlorohydrin, Gas chromatography–mass spectrometry,, Heavy metals, Inductively coupled plasma optic emission spectrometry, Risk

## Abstract

This study aimed to determine the levels of epichlorohydrin (ECH) and some metals in 3 brands of bagged and loose black teas which are widely marketed in Alexandria markets, Egypt, and estimate the acceptable daily intake as a safety indicator. Gas chromatography–mass spectrometry results revealed that ECH levels significantly differed between the tested brands steeped for 2 min and tea bags contained higher levels than in loose teas and the levels increased by increasing the steep time. These levels of ECH in all the tested brands either in tea bags or loose teas were higher than the guideline value of 0.10 μg/L. Also, the results illustrated that the adding of sucrose or washing of bags with deionized water for 1 min significantly decreased the levels of ECH. In addition, inductively coupled plasma optic emission spectrometry (ICP–OES) results illustrated that the levels of Al, Pb, and Cd were too low in infusions compared to the established guidelines. Because of the high consumption of tea in Alexanria city, Egypt, it is necessary to determine the contribution of tea to the daily dietary intake of ECH, Al, Pb and Cd. In case of the bagged teas, the calculated daily intake of ECH was on average about 55.37 times greater than that in loose teas. The consumption of both bagged and loose teas infusion could not pose a risk for population as the health hazard index was < 1. It can be concluded that consumers who prefer to use tea bags should rinse these bags before preparing the tea brewer, and do not increase the steeping time to more than 2 min.

## Introduction

The tea tree (*Camellia sinensis*) is growing in the tropical and subtropical regions under acid soil conditions with significant rainfall of 50 inches/year (Hajiboland 2017). Tea is the most popular beverages after water; with an estimated amount of 18–20 billion tea cups/day are consumed worldwide (Fernandez Caceres et al. 2001). It has an important role for human health because it is considered as a source of essential dietary metals (The Tea Council 2004) and antioxidants (Soni et al. 2015), where the infusion of the tea contains phenolic compounds, sugars, amino acids, alkaloids, minerals, and pigments (Yadav et al. 2020). Consumption of tea infusions may have beneficial health consequences, perhaps contributing to beneficial outcomes in Parkinson's disease, cardiovascular disease, cancer, and immune disorders (Shekoohiyan et al. 2012; Yashin et al. 2015). At the same time, tea infusion can be a reliable dietary source of exposure to contaminants because heavy metals included in tea leaves are differentially extracted into infusions (Karak et al 2017). Economic development and human activities are responsible for environmental issues caused by the accumulation of these contaminants in tea (Li et al. 2014; Lu et al. 2015). Soil, air, and water in which the plants are grown affect level of the contaminants in tea (Valera et al. 1996). Many researchers found excess amounts of metals tend to transfer from the soil to tea (Carr et al. 2003; Han et al. 2006; Wang et al. 2008; Álvarez-Ayuso et al. 2011; Shekoohiyan et al. 2012; Tan and Xiao 2012). Therefore, the drinking of tea infusions containing toxic heavy metals can potentially increase the total body burden of these metals in humans (The Tea Council 2004) and can strongly damage the human health causing carcinogenic, mutagenic, and teratogenic toxicity (Chen et al. 2010; WHO et al. 2012; Hu et al. 2013) since they are nonbiodegradable (Shaheen et al. 2016). For example, the high Al content in the human body has been hypothesized to have possible links with various diseases, such as encephalopathy dementia, and Alzheimer’s disease (Huat et al. 2019; Yokel 2022).

Black tea is the most highly fermented, and popular one, where the enzymatic oxidation of tea polyphenols leads to the formation of chemical compounds which are responsible for the characteristic, aroma, and colour (Balentine et al. 1997). There are many forms of black tea found in the markets, namely stick-shaped, granular, and black tea bags which enclosed in small porous pocket and reinforced with epichlorohydrin (ECH) for wet resistance (Castle et al. 1997; Cao et al. 2006), usually used for a single serving. The use of tea bags was not common until the Second World War, but nowadays, tea bags are widely available and have become an increasingly common choice for consumers. The UK Tea and Infusions Association (2017) reported that the use of teabags in the UK has increased from 5% in 1960 to 96% in 2007 and nowadays about 73% of the population use tea bags over loose tea making the number of tea bags used per day ~14.6 billion (Abebe et al. 2022).

Epichlorohydrin (ECH), or 1-chloro-2,3-epoxypropane is used as wet-strengthening agent in the production of cellulose products, such as tea bag paper to keep the tea bags from falling apart during the preparation of tea infusions. Therefore, tea infusions may contain ECH and/ or its metabolite 3-chloro-1,2-propanediol (3-MCPD) (Bach et al.a 2013). ECH is absorbed following oral administration and distributed within 2–4 h in different organs (Gingell et al. 1985), while its metabolites are much more persistent and tend to accumulate during chronic exposures (Gingell et al. 1987). Although ECH is an FDA-approved chemical, it is classified as a group 2A, a probable carcinogen (IARC, International Agency for Research on Cancer 1999; Göen et al. 2018) and is proven to be harmful to human health (US-FDA 2021), affecting the components of cells (Kucerova et al. 1977; Sram et al. 1983), central nervous system (IARC 1999), reproductive system (US-EPA 1997), and immune system (Wester et al. 1985) as well as causes chromosomal aberrations (Sram et al. 1983; IPCS 1984), carcinomas in different organs (Wester et al. 1985; Göen et al. 2018).

Given the widespread and increasingly prevalent usage of tea bags by consumers, and when teas are brewed in hot water, metals found in its matrix components as well as ECH as wet-strengthening agent in tea bag paper are differentially extracted into infusions, therefore the levels of these contaminants in certain brands of the most consumers' preferred bagged and loose black teas of the same brands widely marketed in Alexandria markets, Egypt, were investigated. Also, the present study aimed to study the effect of preparation conditions of tea infusion (steep time, adding sucrose, and washing the bags) on the levels of ECH and heavy metals in these brands and to estimate the daily intake (EDI) of people as a safety indicator.

## Materials and methods

### Sampling

A total of 60 samples of 3 different brands either of bagged or loose black teas (10, each) were randomly collected from different supermarkets located in Alexandria city, Egypt. Samples were assigned as Ab, Bb, and Cb for tea bags and Al, Bl, and Cl for loose tea. The weights in tea bags were found to be 2.15 ± 0.08, 2.26 ± 0.12, and 2.20 ± 0.09 g /bag for Ab, Bb, and Cb, respectively.

Analytical grade standards for epichlorohydrin (ECH) (1-chloro-2,3-epoxypropane) and its metabolite, 3-monochloropropane-1,2-diol (3-MCPD) were obtained from Merck (USA) with a purity of 99%, while certified HPLC-grades of dichloromethane and methanol, and nitric acid (65%) were purchased from Aldrich (Germany). Ultra-pure deionized water was obtained from a water purification system (PURELAB Option-R, ELGA, UK) and used throughout this study. The stock of certified multi-elements standard (1000 mg/L) was obtained from J.B. Baker Inc. (Phillipsburg, NJ, USA) and used to prepare the working solutions. All other chemicals used in this study were of the highest grade available.

### Determination of ECH by gas chromatography–mass spectrometry (GC–MS)

All glassware and filter papers were rinsed with ultra-pure deionized water and then with certified HPLC-grade of dichloromethane. Dichloromethane was checked for possible ECH and 3-MCPD contamination. All glasses were then dried overnight at 200 °C.

ECH was estimated in empty bags according to the method of Cai and Zou (2010) with slight modifications. Briefly, tea bags from the brands were opened, emptied, and then 100 mL of the boiling deionized water was poured into a beaker containing 3 empty bags of each brand. After 2 min of infusion, the bags were taken out and the steeped infusion was filtered through Whatman filter paper No. 42. When the solution reached room temperature, ECH was extracted with 3 × 10 mL of dichloromethane in the presence of 10 g sodium chloride by using a separatory funnel. The organic layer was separated and evaporated to dryness. The residues were quantitatively transferred to 2 mL clean vials, completed to 1 mL with dichloromethane, and then subjected for analysis by GC–MS. Also, tea infusions from different tea samples were prepared by adding 100 mL of boiling deionized water over either one bag or 3 g (to represent the typical quantity consumed by tea drinkers) of loose tea brands and then stirred with a glass rod for about 1 min to ensure proper wetting and steeped for different times (2, 6, and 10 min). The steeping times were selected according to the tea industry's recommended brew time (1–2, 3–5, and 4–6 min in case of tea bagged and 3–5, 4–6, and 7–9 min in case loose teas for A, B, and C brands, respectively.

The steeped infusion was filtered through Whatman filter paper No. 42 and then extracted as previously described to determine the levels of ECH and 3-MCPD. 3-MCPD was derivatized using Phenyl boronic acid according to Rodman and Ross (1986) and Plantinga et al. (1991).

### Effect of sucrose on the release of ECH

The effect of adding sucrose on the release of ECH either from bagged or loose black teas was carried out by adding 3 or 6 g of sucrose to a beaker containing 100 mL of boiling deionized water and either one tea bag or 3 g of loose tea from each brand. The resulting infusion was stirred with a glass rod for about 1 min to ensure proper wetting and dissolving sucrose, steeped for 2 min, filtered through Whatman filter paper No. 42 and then extracted as previously described to determine the levels of ECH.

### Effect of pre-washing of tea bags on the release of ECH

Tea bags were pre-washed by immersing in 100 mL of deionized water for 1 min and then stirred with a glass rod. The washed tea bags were put in a beaker containing 100 mL of boiling deionized water, stirred with a glass rod for about 1 min to ensure proper wetting, and then steeped for 2 min. The steeped infusion was filtered through Whatman filter paper No. 42 and then extracted as previously described to determine the levels of ECH.

### Gas chromatography–mass spectrometry (GC–MS)

The analysis was carried using gas chromatography (Model GC 450, Varian Inc., The Netherlands) with a mass spectrometer (MS 220.41) equipped with split/splitless injector with electronic pressure and fused silica DB-5and DB-1capillary columns (30 m × 0.25 mm i.d) for the determination of ECH and 3-MCPD, respectively. The MS was run in full scan mode (50–600 m/z) and operated in electron ionization and selected ion monitoring (SIM) modes. The ion energy for electron impact (EI) was kept at 70 eV. MS Workstation Version 6.9.1. was used for data acquisition. For positive identification, both retention time and the presence of three fragment ions (m/z) were considered to provide a high degree of sensitivity and specificity.

In the case of ECH, the temperature inlet was 220 °C, while the oven conditions included an initial temperature of 60 °C, held for 3 min, 30 °C/min ramped to 120 °C, holds for 2 min, followed by 20 °C/min to 180 °C, held for 20 min and then by 15 °C/min ramped to 220 °C. In the case of 3-MCPD, the temperature inlet was 190 °C, while the oven conditions included an initial temperature of 40 °C, held for 3 min, 25 °C/min ramped to 80 °C, hold for 2 min, followed by 30 °C/min to 190 °C, held for 20 min and then by 15 °C/min ramped to 220 °C. Nitrogen was used as a carrier gas at a flow rate of 1 mL/min and 1 μL either of ECH or 3-MCPD was injected.

### Calibration curves

**Fig. 1:**
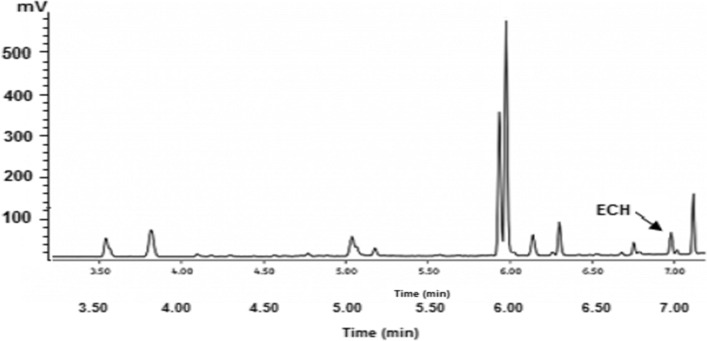
GC–MS chromatogram of tea real sample spiked with 0.10 µg/L of ECH

Calibration experiments were carried out using series concentrations either of ECH or 3-MCPD ranging from 0.10 to 2.0 ng/mL and calibration standards prepared in methylene chloride from the stock solutions (1000 µg/mL in methanol). The amount of ECH or 3-MCPD in each sample was thus calculated based on the slope of the standard curve. Tea extract was spiked with 0.10 and 0.05 µg/L as internal standard solutions of ECH and 3-MCPD, respectively. The. GC–MS chromatogram of a real sample spiked with 0.10 µg/L of ECH is shown in Fig. 1.

### Recovery tests

The samples utilized for the recovery studies were previously analyzed and did not contain residues either of ECH or 3-MCPD. Recovery was carried out at three fortification levels (0.50, 1.0, and 1.50 ng/mL) by adding an appropriate volume of the standard solution to tea-free samples, extracted, and then analyzed as previously described.

### Determination of heavy metals in tea infusions

All glassware and filter papers were rinsed with nitric acid and then with ultra-pure deionized water. Nitric acid was checked for possible trace aluminum, lead, and cadmium contamination. All glasses were then dried overnight at 200 °C.

Tea infusions from different tea samples were prepared by adding 100 mL of boiling deionized water over either one bag or 3 g (to represent the typical quantity consumed by tea drinkers) of loose tea brands and, with a glass rod for about 1 min to ensure proper wetting and then steeped for steeped for 2, 6, and 10 min. The steeped infusion was filtered under vacuum through Whatman filter paper No. 42, evaporated until a clear solution was obtained, allowed to cool down, and then transferred to a 25 mL volumetric flask containing 5 mL of concentrated nitric acid. The mixture was vigorously mixed, made up with deionized water to 25 mL and stored in a polypropylene screw-capped bottle for analysis. Aluminum (Al), lead (Pb), and cadmium (Cd) were analyzed by the inductively coupled plasma optic emission spectrometry (ICP-OES EOP Spectroacros, Model Varian Vista-MPX, USA) according to the method of US-EPA (1994). The operating conditions were adjusted according to the standard guidelines of the manufacture. The purity of argon as carrier gas was 99.999%, with a flow rate of 0.30 L/min for supplementary and 19 L/min for coolant flow. The power level was adjusted to 1100 KW. The wavelengths (nm) were 308.215, 220.353, and 226.502 nm for Al, Pb, and Cd, respectively. Blank solutions were prepared in a similar way without tea bags or loose tea under identical conditions and the average signal was subtracted from analytical signals of tea samples. For correction of the matrix interference, an organic tea sample that is completely free of toxic heavy metals was used. This base tea was pretreated like the tested samples and used as a sample matrix. Analytical calibration, Intermediate, and spiking mixture standard solutions were prepared directly from the certified metal standard solution (1000 mg/L) by diluting with 2% of HNO_3_ for obtaining different concentrations, and the concentration of each sample in the mass of the sample taken was calculated. Concentrations of metals were quantified using seven-point external calibration curves within the concentration ranging from 0.00 to 4.00 mg L^−1^.

### Statistical analysis

All data were calculated as mean ± standard deviation (SD) and analyzed using analysis of variance technique (ANOVA). A probability of 0.05 or less was considered significant.

## Results and discussion

### Methods validation

The linearity of calibration curves was evaluated based on the correlation coefficients (R^2^), where the R^2^ values > 0.989 in case of ECH and 3-MCPD, while in case of the tested metals the R values were > 0.963. Recoveries and precision of the method (expressed as the relative standard deviation, RSD) were calculated as percentages, while limits of detection (LOD) and quantitation (LOQ) were calculated from the signal-to- noise ratios obtained by analyzing unspiked samples (n = 10), where LOD and LOQ were determined as the amount of a ECH, 3-MCPD, Al, Pb or Cd giving a signal-to-noise ratio of 3 and 10, respectively. The methods of ECH and 3-MCPD analysis were validated by determining LOD, LOQ, recovery and RSD at different levels of the fortification. For ECH and 3-MCPD, the recoveries, RSD, LOD, and LOQ were 96–106% and 2–6%, 0.08 and 0.11 µg/L and 0.26 and 3.05 µg/L, respectively.

In case of metals, the LOD values were 0.10, 1.80, and 0.08 µg/L, while LOQ values were 0.31, 4.90, and 0.25 µg/L for Al, Pb, and Cd, respectively, with percentages of recovery ranged from 93 to 110 and RSD of 3–5%.

### Effect of steep time on the levels of ECH

To study the effect of ECH on the health of tea consumers, it is important to understand how steep time alters the levels of ECH in tea infusion prepared under laboratory experiments in a similar manner as a consumers do. Data in Table 1 illustrate that significant differences between the levels of ECH in the bags of the three tested brands steeped for 2 min were observed, where the mean levels were 1.82, 3.06, and 3.48 µg/L for A, B, and C brands, respectively. Also, the results indicated that tea bags contained high significant levels of ECH compared with that found in loose tea in all the tested brands and these levels increased with increasing the steep time either for bagged or loose black teas. The detected levels of ECH in tea bags ranged from 1.68–4.94, 2.29–9.31, to 3.22–11.40 µg/L, while the levels in loose teas ranged from 0.36–0.49, 0. 42–0.52, to 0. 52–0.65 µg/L in infusions of tea bags of brands A, B, and C, respectively, when the tea was steeped for different time intervals (2–10 min), indicating that brand A either of tea bags or loose tea was found to contain less levels of ECH followed by brand B and then brand C.

**Table 1:** Levels (µg/L) of ECH in infusions of bagged and loose black teas steeped for different times

Brand	Bags	Tea bags			Loose teas		
	2 min	2 min	6 min	10 min	2 min	6 min	10 min
A	1.82 ± 0.10^Ca^	1.68 ± 0.03^ Da^	2.77 ± 0.12^Ba^	4.94 ± 0. 02^Aa^	0.36 ± 0.04^Ha^	0.42 ± 0.05^ Ga^	0.49 ± 0.06^Fa^
B	3.06 ± 0.10^Cb^	2.29 ± 0.31^Db^	4.52 ± 0.29^Bb^	9.31 ± 0. 21^Ab^	0.42 ± 0.0^Fa^	0.45 ± 0.06^Fa^	0.52 ± 0.05^Ea^
C	3.48 ± 0.15^Cc^	3.22 ± 0.47^Dc^	4.61 ± 0.22^Bb^	11.40 ± 0.31^Ac^	0.52 ± 0.05^ Gb^	0.61 ± 0.07^Fb^	0.65 ± 0.07^Fb^

Data are expressed as mean ± SD (n = 10)

Means followed by the same small letters within each column do not significantly different from each other (*p* ≤ 0.05)

Means followed by the same capital letters within each raw do not significantly different from each other (*p* ≤ 0.05)

Because ECH has recently been recognized as a potential carcinogen compound (NIOSH 2014), therefore many countries have begun imposing limits on the amount of ECH allowable in drinking water of 0.1 μg/L (Council Directive 1998) or zero in order to avoid health risks (US-EPA 2010). In the present study, the levels of ECH in all the tested brands either in tea bags or loose teas were higher than the guideline values (Council Directive 1998; US-EPA 2010).Therefore, these bags are not totally safe for human and an innovative design of non-toxic tea bags is required (Abebe et al. 2022).

Data in Table 2 illustrate that 3-MCPD was not detected either in tea bags or loose teas of brand A. However, the levels of 3-MCPD increased with increasing the steep times when tea bags of brands B and C were steeped for 2–10 min with mean values ranged from 0.03–0.05 to 0.04–0.06 µg/L, respectively. However, non-detected levels of 3-MCPD were found when the loose teas either of B or C brands steeped for 2 min, whereas at the levels ranged from 0.01 to 0.02 µg/L for both brands when the steep timed ranged from 6 to 10 min. In general, the tea brewing water used is ranging from 90 to 100 °C, and most tea bags may leach toxic compounds or even undergo thermal degradation (Bach et al. 2013) to form 3-MCPD and the rate of hydrolysis increases sevenfold when the temperature is raised to 40 °C (von Pringer 1980). According to Codex Alimentarius, 3-MCPD is formed at high temperature and should be present in low amounts as 3-MCPD migrates to beverages from packaging material and is highly carcinogenic (Bassi et al 2020). In the present results, water was boiled at 100 °C to prepare the tea infusion with releasing amounts of ECH and 3-MCPD. Therefore, we can certainly presume that ECH leached from teabags into infusions may pose a risk for human consumption and an innovative design of non-toxic tea bags is required (Abebe et al. 2022).

**Table 2:** Levels (µg/L) of 3-MCPD in infusions of bagged and loose black teas steeped for different times

Brand	Tea bags			Loose teas		
	2 min	6 min	10 min	2 min	6 min	10 min
A	ND^Aa^	ND^Aa^	ND^Aa^	ND^Aa^	ND^Aa^	ND^Aa^
B	0.03 ± 0.001^Ab^	0.04 ± 0.001^Bb^	0.05 ± 0.001^Cb^	ND^Da^	0.01 ± 0.003^Eb^	0.02 ± 0.009^AEb^
C	0.04 ± 0.001^Ab^	0.05 ± 0.002^ABb^	0.06 ± 0.004^Bb^	ND^CAa^	0.01 ± 0.005^Db^	0.02 ± 0.006^Db^

Data are expressed as mean ± SD (n = 10)

Means followed by the same small letters within each column do not significantly different from each other (*p* ≤ 0.05)

Means followed by the same capital letters within each raw do not significantly different from each other (*p* ≤ 0.05)

ND means non-detected

### Effect of adding sucrose on the levels of ECH

Data in Table 3 represent the effect of sucrose on the levels of ECH in infusions of tea bags and loose teas steeped for 2 min. It was found that there were significant differences between the three tested brands and between using and non-using sucrose regarding the levels of ECH in tea fusions either for tea bags or loose teas. However, non- significant differences between the using of 3 or 6 g of sucrose on the levels of ECH in tea infusions either of tea bags or loose tea were recorded. When 3 and 6 g of sucrose were added to the infusions of tea bags, the levels of ECH was reduced by 13 and 14%, 15 and 17%, and 20 and 21% for A, B, and C brands, respectively. In case of loose teas, the percentages of reduction were 11 and 14% and 22 and 25%, and 18 and 20% when 3 and 6 g of sucrose were added to the infusions of A, B, and C brands, respectively.

**Table 3:** Effect of sucrose on the levels (µg/L) of ECH in infusions of bagged and loose black teas steeped for 2 min

Brand	Tea bags			Loose teas		
	Without sucrose	3 g sucrose	6 g sucrose	Without sucrose	3 g sucrose	6 g sucrose
A	1.68 ± 0.03^Ba^	1.46 ± 0.07^Aa^	1.44 ± 0.12^Aa^	0.36 ± 0.07^Ca^	0.32 ± 0.04^ Da^	0.31 ± 0.02^ Da^
B	2.29 ± 0.31^Bb^	1.95 ± 0.02^Ab^	1.90 ± 0.42^Ab^	0.54 ± 0.06^Cb^	0.42 ± 0.06^Db^	0.40 ± 0.06^Db^
C	3.22 ± 0.47^Ac^	2.59 ± 0.26^Bc^	2.53 ± 0.31^Bc^	0.62 ± 0.05^Cc^	0.51 ± 0.07^Dc^	0.41 ± 0.08^Dc^

Data are expressed as mean ± SD (n = 10)

Means followed by the same small letters within each column do not significantly different from each other (*p* ≤ 0.05)

Means followed by the same capital letters within each raw do not significantly different from each other (*p* ≤ 0.05)

### Effect of washing of tea bags on infusions ECH levels

It was found that when tea bags were washed first with deionized water for 1 min and then the tea bags steeped for 2 min. Significant differences between washed and non-washed tea bags with water regarding the levels of ECH (Table 4). As it was expected from the high-water solubility of ECH which equals 65.90 g/L at 25 °C (NCBI 2022), ECH was significantly removed by water by 93, 93, and 94% for A, B, and C brands, respectively.

**Table 4:** Effect of washing process on the levels (µg/L) of ECH in infusions of tea bags steeped for 2 min

Brand	Without washing	After washing	% of removing
A	1.68 ± 0. 03^Aa^	0.12 ± 0.01^Ba^	92.86
B	2.29 ± 0.31^Ab^	0.16 ± 0.01^Bba^	93.01
C	3.22 ± 0.047^Ac^	0.20 ± 0.02^Bb^	93.79

Data are expressed as mean ± SD (n = 10)

Means followed by the same small letters within each column do not significantly different from each other (*p* ≤ 0.05)

Means followed by the same capital letters within each raw do not significantly different from each other (*p* ≤ 0.05)

The present study recommend to use of special type of packaging material is important parameter which needs the attention of scientific community to find out alternatives of tea bags which should be eco-friendly, nontoxic, and most importantly low cost in nature (Bassi et al 2020).

### Mineral analysis of tea infusions

Tea is the second one after water as the most popular beverage and the determination of heavy metals in the infusions of bagged and loose black teas is therefore important for estimating the daily intake of people as a safety indicator. The present study was carried out to investigate the effect of steep time and tea type on the release of Al, Pb, and Cd in the infusions of bagged and loose black teas of the same brand. The levels of toxic elements in this study for Al, lead Pb, and cadmium Cd are outlined in Tables 5, 6, and 7.

**Table 5:** Levels (µg/L) of Al in infusions of bagged and loose black teas steeped for different times

Brand	Tea bags			Loose teas		
	2 min	6 min	10 min	2 min	6 min	10 min
A	0.012 ± 0.001^Aa^	0.013 ± 0.0002^Aa^	0.014 ± 0.002^Aa^	0.011 ± 0.001^Aa^	0.014 ± 0.002^ABa^	0.017 ± 0.003^Ba^
B	0.019 ± 0.002^Ab^	0.021 ± 0.0002^Bb^	0.021 ± 0.002^Bb^	0.014 ± 0.002^Cb^	0.017 ± 0.002^Dab^	0.019 ± 0.0001^Aab^
C	0.021 ± 0.004^Ac^	0.024 ± 0.002^Bc^	0.027 ± 0.001^Cc^	0.016 ± 0.002^Dc^	0.019 ± 0.004^Ab^	0.021 ± 0.003^Ab^

Data are expressed as mean ± SD (n = 10)

Means followed by the same small letters within each column do not significantly different from each other (*p* ≤ 0.05)

Means followed by the same capital letters within each raw do not significantly different from each other (*p* ≤ 0.05)

**Table 6:** Levels (µg/L) of Pb in infusions of bagged and loose black teas steeped for different times

Brand	Tea bags			Loose teas		
	2 min	6 min	10 min	2 min	6 min	10 min
A	0.01 ± 0.001^Aa^	0.01 ± 0.001^Aa^	0.01 ± 0.001^Aa^	0.01 ± 0.001^Aa^	0.01 ± 0.001^Aa^	0.01 ± 0.001^Aa^
B	0.01 ± 0.001^Aa^	0.01 ± 0.001^Aa^	0.01 ± 0.001^Aa^	0.01 ± 0.001^Aa^	0.01 ± 0.001^Aa^	0.01 ± 0.001^Aa^
C	0.02 ± 0.004^Ab^	0.02 ± 0.001^Ab^	0.02 ± 0.003^Ab^	0.01 ± 0.001^Ba^	0.01 ± 0.001^Ba^	0.01 ± 0.001^Ba^

Data are expressed as mean ± SD (n = 10)

Means followed by the same small letters within each column do not significantly different from each other (*p* ≤ 0.05)

Means followed by the same capital letters within each raw do not significantly different from each other (*p* ≤ 0.05)

**Table 7:** Levels (µg/L) of Cd in infusions of bagged and loose black teas steeped for different times

Brand	Tea bags			Loose teas		
	2 min	6 min	10 min	2 min	6 min	10 min
A	0.10 ± 0.001^Aa^	0.10 ± 0.001^Aa^	0.10 ± 0.001^Aa^	0.10 ± 0.001^Aa^	0.10 ± 0.001^Aa^	0.10 ± 0.001^Aa^
B	0.10 ± 0.001^Aa^	0.10 ± 0.001^Aa^	0.10 ± 0.001^Aa^	0.10 ± 0.001^Aa^	0.10 ± 0.001^Aa^	0.10 ± 0.001^Aa^
C	0.10 ± 0.001^Aa^	0.10 ± 0.001^Aa^	0.10 ± 0.001^Aa^	0.10 ± 0.001^Aa^	0.10 ± 0.001^Aa^	0.10 ± 0.001^Aa^

Data are expressed as mean ± SD (n = 10)

Means followed by the same small letters within each column do not significantly different from each other (*p* ≤ 0.05)

Means followed by the same capital letters within each raw do not significantly different from each other (*p* ≤ 0.05)

The results revealed significant differences in Al concentrations among the three tested brands either of tea bags or loose tea (Table 5). Also, the results showed that tea bags contained levels of Al higher than that found in loose tea when steeped for 2–10 min and brand C contained higher levels of Al followed by brand B and then brand A. Also, the results indicated that tea bags contained high significant levels of Al compared with that found in loose teas in all the tested brands and these levels increased with increasing the steep time. The detected levels ranged from 0.012–0.014, 0.019–0.021, to 0.021–0.027 µg/L in case of tea bags, while the levels in loose teas ranged from 0.011–0.017, 0.014–0.019, to 0.016–0.021 µg/L in infusions of brands A, B, and C, respectively, when the tea was steeped for different time intervals (2–10 min), indicating that brand A either of tea bags or loose teas was found to contain less levels of Al followed by brand B and then brand C. The amount of Al released either from tea bags or loose tea in the infusion process was below the maximum values set by the guidelines. Many investigators collected tea samples from major tea-growing countries and found that all tea samples released Al during a standard infusion period (Wong et al. 1998; Shokrzadeh et al. 2008; Fung et al. 2009). Tea leaves and leaf infusions were found to contain high concentrations of Al which associated with the different production processes, quality of the tea, industrial activity and the use of pesticides and fertilizers (Rao 1994; Atafar et al. 2010; Parviz et al. 2015). The present results are in parallel with the results of Fung et al. (2009) who found that all the tested tea brands released Al during the tested infusion periods and the infusion time influences the contents of Al, where the solubility of Al in boiling water showed that the transfer of Al to the brew was positively correlated with the infusion time (Ghoochani et al. 2015). Also, Al was found to be the most abundant element in the leaf and bagged tea samples (Polechońska et al. 2015), where tea is considered as an Al-hyperaccumulator, accumulating both Al and other metals (Carr et al. 2003; Mehra and Baker 2007; Karak and Bhagat, 2010). Unfortunately, very small amounts of Al are needed to produce neurotoxicity, and experimental study showed that chronic Al intoxication reproduced neuropathological hallmarks of Alzheimer's disease (Tomljenovic 2011).

Data presented in Table 6 illustrate that Pb concentrations of the tea bags of brand C were significantly higher than the values for the tea bags of brands A and B, where the recorded levels were 0.02, 0.01, and 0.01 µg/L, respectively. Our results are in agreement with Rashid et al. (2016) who reported that Pb, Cd, and Cr concentrations in the tea samples were below the maximum values set by the guidelines. Also, the present results are in line with that reported by Shen and Chen (2008) who determined a mean Pb content of 0.01 mg/Kg in green tea samples from Taiwan, and lower than detected in several studies (Zheng et al. 2014; Li et al. 2014; de Oliveira et al. 2018). These results revealed that Pb concentration in the examined tea samples could to be negligible. However, Pb showed minimum levels of 1.41 μg/g in several commercially available brands of tea in Egypt (Soliman 2016). Also, the present results showed that steeping times had no effect on Pb content either for the tea bags or loose tea. However, infusion time was found to influence Pb, Cd, and As contents in infused tea (Shekoohiyan et al. 2012; Parviz et al 2015). The differences between the results may be due to differences in the types of samples or kinds of contamination at each sample’s origin (Parviz et al 2015).

The levels of Cd in infusions either of tea bags or loose teas steeped for different times were 0.10 µg/L indicating that the steep time had no effect on Cd levels (Table 7). In the present study, the detected Cd levels are much lower than the literatures (Zheng et al. 2014; Li et al. 2014; Deka et al. 2021). Fortunately, the obtained concentrations of Al, Pb, and Cd were too low in infused samples compared to established guidelines (WHO 1998 and 2007; EU 2020). However, Shekoohiyan et al. (2012) investigated that infusion time influenced Cd content in infused tea. Although these levels of metals do not produce toxicity in healthy individuals having normal absorption and excretion system, they could be potentially harmful in the case of renal disorder (Jalbani et al. 2007). The variation in the levels of these metals depends on many factors such as leaf age, plant genetics and cultivars, the method of sample preparation, weather, the use of agrochemicals, industrial activities, and type of soil, (Parviz et al. 2015; Tao et al. 2021). When large amounts of fertilizers have been applied to obtain a high yield of tea (Hajiboland 2017), high levels of metals will be detected because tea plants are considered as accumulator species for metals (Poschenrieder et al. 2008; Polechońska et al. 2015). The results of the present study are supported by many studies, where tea bags were found to contain higher levels of metals Al, Mn, and Pb because tea bags often contain older leaves and low-cost tea materials (Shu et al. 2003; Wong et al. 2003; Yemane et al. 2008; Karak et al. 2010). Also, the present results are in parallel with the results of Polechońska et al. (2015) who hypothesized that bagged black tea contained higher amounts of trace metals than leaf tea of the same brand and that the trace metal content in infusion made from bagged tea was higher than made from leaf tea. It may be associated with different production processes and the quality of the tea. Different contents of elements in different types of tea (e.g., granular tea leaves, powder, and tea bags) were associated with the different processing methodology (Kumar et al. 2005), where a high content of these elements in dry tea is due to the lower quality of bagged teas (Polechońska et al. 2015). This is of importance to the consumer, as the use of bagged teas is very popular worldwide (Mehra and Baker 2007).

### Estimated daily intake of ECH, Al, Pb and Cd

Although tea has benefits for human health (Ambadekar et al 2012; Chen et al. 2012), the consumption of tea may provide also a significant contribution for intake of contaminants in the human body. Therefore, the determination of these contaminants in their infusion is important for estimating the daily intake of people, considered as a safety indicator. The total body burden of contaminants in the human body due to the consumption of tea depends on the total content in tea, characteristics of the water used for brewing, a fraction of the total content extracted to the infusion, and bioavailability from the beverage (Pękal et al. 2013; Göen et al.a 2017). Thus, knowing the content of ECH, Al, Pb, and Cd in tea is important because of their adverse effects on human health let us to assess the estimated daily intake (EDI) through drinking tea with the acceptable daily intakes (ADI) established by the guidelines (Table 8). The ADI of a contaminant is the amount of that contaminant that can be ingested daily by a human being during an entire lifetime without appreciable risk to the health. Egypt is the largest market for tea in the Middle East and North Africa area and ranked in the top ten global consumers, with consumption in 2003 of 77,400 tones with only small exports. This put consumption at 1.06 kg/person/year in 2003, or 3–5 cups/day (FAO, 2005; Alboghdady and Allokka, 2013). Because tea is an indispensable part of everyday life for many people in in Alexandria city with high consumption, it is necessary to determine the contribution of tea to the daily dietary intake of ECH, Al, Pb and Cd to ensure that public health is maintained.

**Table 8:** Estimated daily intakes (EDIs) and ADIs of epichlorohydrin, Al, Pb and Cd found in infusions of bagged and loose black teas

Contaminant	ADI (mg/Kg/day) ^a^	Tea bags EDI (mg/Kg/day)	Hazard index (EDI/ADI, %)	Loose teas EDI (mg/Kg/day)	Hazard index (EDI/ADI, %)
ECH	0.15^a^	1.03 × 10^–2^	6.87 × 10^–2^	1.86 × 10^–4^	1.24 × 10^–5^
3-MCP	–	1.00 × 10^–6^	–	–	–
Al	7.5^b^	2.48 × 10^–8^	3.31 × 10^–9^	1.95 × 10^–7^	2.60 × 10^–8^
Pb	10^c^	1.90 × 10^–7^	1.90 × 10^–8^	1.43 × 10^–7^	1.43 × 10^–8^
Cd	1^d^	1.43 × 10^–6^	1.43 × 10^–6^	1.43 × 10^–6^	1.43 × 10^–6^

^a^US-EPA (1985)

^b^According to WHO (1998)

^c^According to WHO (2007)

^d^EU (2020)

Data in Table 8 compare the estimated contribution of tea consumed to the intake of these contaminants with the ADI (US-EPA 1985; WHO, 1998, 2007; EU 2020). No ADI for 3-MCP has been published. The EDIs for ECH were 1.03 × 10^–2^ and 1.86 × 10^–4^ mg/Kg/day), while the hazard index, HI (EDI/ADI, %) were 6.87 × 10^–2^ and 1.24 × 10^–5^, for tea bags and loose teas, respectively. In case of heavy metals, Cd showed the maximum daily intakes either in the tea bags or loose teas (1.43 × 10^–6^ mg/Kg/day). In case of the bagged teas, the calculated daily intake of ECH was on average about 55.37 times greater than that in loose teas. The EDI_s_ for the three tested metals ranged from 2.48 × 10^–8^ to 1.43 × 10^–6^ to 1.43 × 10^–7^ to 1.43 × 10^–6^ mg/Kg/day, while the hazard index ranged from 3.31 × 10^−9^to 1.43 × 10^–6^ to 2.60 × 10^–8^ to 1.43 × 10^–6^ for tea bags and loose tea, respectively. Thus, lifetime consumption of these brands of teas could not pose risks for the Alexandrian population as the indices for all the levels were less than one (US-EPA 1989). The HI values are usually used to estimate the possible non-carcinogenic effects of heavy metals on human health via the consumption of fruits, vegetables, honeys and beverages (Shaheen et al. 2016; de Oliveira et al. 2018; Peng et al. 2018; Osman et al. 2021). The HI values for exposure to As, Cd, Cr, and Pb in three types of teas (green, black, and oolong) collected from Taiwan were also less than one as found in the present study, indicating no risk to human health (Shen and Chen, 2008). Although the health risk of heavy metals ingestion via tea intake was relatively small, more comprehensive investigation for dietary structure and heavy metal contents in food are needed for health risk assessment.

The present results confirmed that Al and Pb concentrations were much lower than those concentrations set by WHO (1998). The present findings are in parallel with many researchers who reported that the exposure to Al, Cd, Hg, As, Sb, Cr, and Ni do not represent a hazard to general health via infusion intake of tea leaves (Hayacibara et al. 2004; Zhang et al. 2020; Tao et al. 2021). However, possible health risks of heavy metals in tea were reported and the exposure for long-term to these toxic metals resulting in their accumulation in human body and increase the total body burden (de Oliveira et al. 2018; Zhang et al. 2018a, b; Sun et al. 2019). Each country sets its own allowable limit for Pb concentration in tea leaves which is 5, 20, and 2 mg/Kg in Europe, Japan, and China, respectively. Recent increases in the Pb concentration of commercial tea supplies have caused concern to both consumers and producers. Therefore, it is recommended to select specific fertilizer to immobilize the transfer and accumulation of heavy metals in tea in order to decrease and/or avoid risks by these metals (Tao et al. 2021).

## Conclusion

There is growing interest regarding the components of the tea bags that may confer health problems with regular consumption. In this study, we have investigated the levels of ECH, 3-MCPD, Al, Pb, and Cd in infusions of bagged and loose black teas of the same brand to assess the consumer exposure as they would be consumed habitually by millions of individuals in Alexandria city, Egypt every day. Also, the present study aimed to investigate the effect of steep time, adding sucrose, and washing the bags on release of these ECH into the infusions of these brands and to estimate the daily intake of people as a safety indicator. A comparison of the current results with the established standard values indicated that all the levels of the investigated metal were within standard ranges. Generally, non-essential elements such as Al, Pb, and Cd and ECH and 3-MCPD contents of tea bags were found to be higher than those of loose tea infusions. Because of the better extractability of metals from tea materials that had been crushed more thoroughly, the infusions of bagged tea contained higher levels of Al in relation to the infusions of loose teas. These levels of such contaminants were below the limits of WHO and EU standards and do not produce risk in Alexandrian humans. Although low daily intakes of the tested contaminants show that the consumption of these brands of tea either of bagged or loose are not dangerous for human health, it is essential to have a good tea plant quality control. Moreover, water composition plays an important role in chemical extraction from tea and strongly determines the composition of tea infusion. In the present study, tea infusions were prepared using ultra-pure deionized water. However, tea for consumers will be prepared with water from tap water and hence contains different elemental concentrations, and this may affect elemental concentrations in the tea infusion.

In conclusion, the present investigation can serve as an eye opener to consumers to know the optimum conditions (steep time, amount of sucrose, the prewashing of bags before using) to prepare tea infusion in order to reduce the potential health risks. Due to the rise in demand for tea bags for tea preparation, the tea infusion parameters have to be optimized. For example, Consumers who prefer to use tea bags should rinse these bags before preparing the tea brewer, and do not increase the steeping time to more than 2 min. On the basis of the above findings, the results recommend the need for regular monitoring of a greater number of tea samples for long periods of time to get deeper knowledge about the fulfillment of teas marketed in Alexandria markets to prevent and/or reduce health problems. This study has provided important information on contamination of teas from Alexandria City for the first time and can be used as a reference for future in depth studies and as a starting point to regulate many other metals in teas.

## Data Availability

The data and material presented in this study are included within the article and are available on request from the corresponding author.

## Abbreviations

Al: Aluminum
ADI: Acceptable daily intake
Cd: Cadmium
ECH: Epichlorohydrin
EDI: Estimated daily intake
EDI/ADI: Hazard index
GC–MS: Gas chromatography–mass spectrometry
ICP–OES spectrometer: Inductively coupled plasma optic emission spectrometry
Pb: Lead

## Declarations

### Clinical trials registration

This study does not contain any studies with clinical trials performed by any of the authors.

### Plant reproducibility

This article does not contain any studies with Plant Reproducibility performed by any of the authors.
